# Updates on immunological mechanistic insights and targeting of the oral lichen planus microenvironment

**DOI:** 10.3389/fimmu.2022.1023213

**Published:** 2023-01-09

**Authors:** Xiaoting Deng, Ying Wang, Lu Jiang, Jing Li, Qianming Chen

**Affiliations:** State Key Laboratory of Oral Diseases, National Clinical Research Center for Oral Diseases, Chinese Academy of Medical Sciences Research Unit of Oral Carcinogenesis and Management, West China Hospital of Stomatology, Sichuan University, Chengdu, Sichuan, China

**Keywords:** oral lichen planus, immune microenvironment, immune cells, chemokine, immune therapy

## Abstract

Oral lichen planus (OLP) is a chronic immune inflammatory disease that is an oral potentially malignant disorder (OPMD), occurs in the oral mucosa and affects approximately 0.5% to 4% of the general population. There are usually five types of OLP: reticular/papular, plaque-like, atrophic/erythematous, erosive/ulcerative, and bullous. Furthermore, the chance of causing oral squamous cell carcinoma (OSCC) is 1.4%. Although the etiology of OLP is still unknown, accumulating evidence supports that immune dysregulation may play a vital role in the pathogenesis of OLP, especially the massive production of various inflammatory cells and inflammatory mediators. In this review, we focus on the relationship between OLP and its immune microenvironment. We summarize current developments in the immunology of OLP, summarizing functional cell types and crucial cytokines in the OLP immune microenvironment and the underlying mechanisms of key signaling pathways in the OLP immune microenvironment. We highlight the application potential of targeted immune microenvironment therapy for OLP.

## 1 Introduction

Oral lichen planus (OLP) is a chronic inflammatory condition that occurs in the oral mucosa and affects approximately 0.5% to 4% of the general population, and its malignant transformation rate is 1.4% (1, 2). Histopathologically, this lesion is characterized by lymphocyte band-like invasion of the lamina propria and basal keratinocyte destruction. The etiology of OLP may be primarily related to immune, genetic, infection, psychiatric, endocrine, microcirculation disorders and trace element deficiency. Mounting evidence supports immunological processes playing critical roles in the pathogenesis of OLP, especially the massive production of various inflammatory cells and inflammatory mediators caused by immune dysregulation (3). It is currently suggested that persistent aggregation of T lymphocytes leads to a chronic inflammatory response to OLP. A range of cells and cytokines in the oral mucosa are involved in the development of OLP by affecting T lymphocyte-mediated basal cell liquefaction and epithelial keratinocyte apoptosis. Recently, there has been awareness of the association of chronic inflammation with various epithelial malignancies, such as chronic esophagitis-associated esophageal adenocarcinoma of esophagitis and chronic inflammatory enteritis-associated bowel cancer (4–7). Existing evidence suggests that the chronic inflammatory process per se is able to provide a cytokine-based microenvironment that is able to influence cell survival, growth, proliferation, differentiation and movement, hence the microenvironment contributes to cancer initiation, progression, invasion and metastasis (7, 8). The bases of OLP malignant transformation might be found in the OLP subepithelial inflammatory microenvironment, which presents activated inflammatory cells and cytokine networks that may act to promote squamous tumorigenesis. The production of tumor necrosis factor, macrophage inhibitory factor, matrix metalloproteinases (MMPs), chymase, interleukin 4 and 6 by macrophages, mast cells, fibroblasts and T-lymphocytes may lead to angiogenesis, degradation and remodeling of the extracellular matrix, thus ultimately influencing oral epithelial cell growth, survival, migration, apoptosis, and neoplastic transformation and ultimately promoting tumor initiation (9). The study of the OLP immune microenvironment greatly enriched our understanding of OLP and opened a broad field for the treatment of this disease. In this paper, we summarize current developments in the immunology of OLP.

## 2 OLP immune microenvironment

The immune microenvironment is a complex dynamic network system composed of many kinds of cells and immune molecules (10). Various injury factor stimulations can lead to inherent immune cell and immune cell type and function changes, resulting in local immune homeostasis and microenvironment changes and leading to chronic disease. The pathogenesis of OLP is currently controversial, but substantial evidence suggests that immune dysregulation plays a key role (3). Potential mechanisms related to immunopathogenesis may include antigen-specific cell-mediated immune responses, nonspecific mechanisms, autoimmune responses, and humoral immunity (11). Regardless of these immunopathologies, the immune microenvironment plays a crucial role. Alternatively, the immune microenvironment may influence the characteristics of the immune response, disease severity, and duration of OLP. The cellular and cytokine networks activated in the immune microenvironment may underlie OLP carcinogenesis (9).

**Figure 1:**
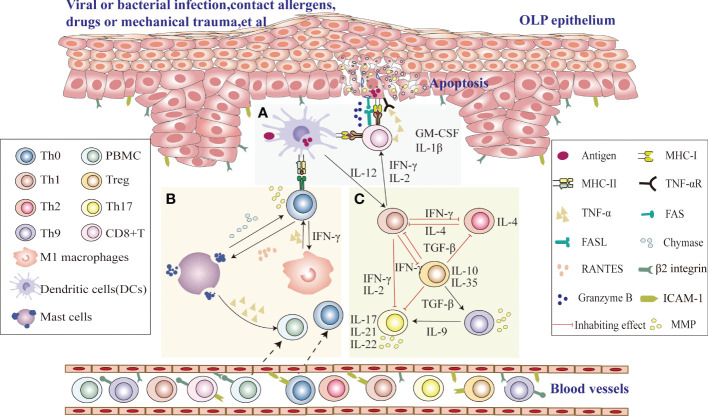
Possible role of the immune microenvironment in OLP pathogenesis **(A)** Upon stimulation with viruses, bacterial infection, drugs or mechanical damage, CD8^+^ T cells identify antigen-associated MHC class I molecules on target cells (damaged keratinocytes or DCs) that activate CD8^+^ cytotoxic T cells directly, and DCs in OLP lesion tissues process antigen-derived peptides and present them together with MHC class II molecules to CD4^+^ helper T cells, which recruit CD8^+^ T cells to subepithelial regions and activate CD8^+^ T cells *via* IL-2 and IFN-γ. Activated CD8^+^ T cells cause keratinocyte apoptosis, which may be due to FAS-FAS ligand interactions between keratinocytes and cytotoxic T cells, cytotoxic T cells producing granzyme B in keratinocytes through perforin-induced membrane pores, and TNF-α secreted by cytotoxic T cells binding to the TNF-α receptor on the keratinocyte surface, leading to the occurrence of OLP. **(B)** DCs in OLP lesion tissues process antigen-derived peptides and present them together with MHC class II to CD4^+^ helper T cells. Local production of IFN-γ secreted by Th1 cells activates proinflammatory type M1 macrophages. The production of TNF-α by type M1 macrophages can indirectly increase the disruption rate of the basement membrane by MMP-9 produced by T cells. RANTES secreted by T cells may attract mast cells into the developing OLP lesion and subsequently stimulate mast cell degranulation. Degranulating mast cells in OLP release TNF-α and chymase. TNF-α promotes inflammatory cell recruitment, and chymase directly or indirectly increases the disruption rate of the basement membrane by T-cell-secreted MMP-9. Thus, periodic interactions between T cells and mast cell secretion may be responsible for the long-term and chronic duration of the disease. **(C)** Th1 cytokines in local OLP lesions can aggravate the severity of the disease by the production of IFN-γ, IL-2 and TNF-α and thus negatively regulate Th17 differentiation and downregulate the immunosuppressive effect of TGF-β1. However, Th2 cells can maintain chronic disease by producing IL-4. The secretion of IL-17, IL-21, and IL-22 by Th17 cells may increase the inflammatory response. Furthermore, Th17 cells may also lead to an impaired epithelial basement membrane through the release of MMPs. Th9/IL-9 can exacerbate the occurrence of OLP by directly increasing Th17-cell levels or indirectly upregulating MMP9 levels.

Since the immune microenvironment plays an important role in the pathogenesis of OLP, a thorough study of the immune microenvironment signaling pathway in OLP is critical for us to seek the correct therapeutic targets (**Figure 1**). Upon stimulation with viruses, bacterial infection, drugs or mechanical damage, MHC class II molecules and antigen binding on the surface of immature DCs in OLP lesion tissues are presented to CD4^+^ helper T cells. Their secreted interleukin-12 (IL-12) induces cytokine release from CD4^+^ T cells, including interferon gamma (IFN-γ) and IL-2, which recruit CD8^+^ T cells to subepithelial regions, activate CD8^+^ T cells, and activate proinflammatory type M1 macrophages, eventually leading to the onset of chronic inflammation (12). Then, CD8^+^ T lesion cells cause apoptosis when in contact with diseased keratinocytes with the corresponding antigens, which may be due to FAS-FAS ligand interactions between keratinocytes and cytotoxic T cells, cytotoxic T cells producing granzyme B into keratinocytes through perforin-induced membrane pores, and TNF-α secreted by cytotoxic T cells binding to the TNF-α receptor on the keratinocyte surface, leading to the occurrence of OLP (13). Th1 cells, which secrete the specific cytokines IFN-γ, IL-2 and TNF-α, recruit monocytes to lesions to differentiate into proinflammatory M1 macrophages, intensifying inflammation by secreting proinflammatory cytokines that upregulate cell adhesion molecules on the surface of endothelial cells and keratinocytes and induce T-cell chemokine expression (RANTES) to promote inflammatory cell recruitment (14, 15). Upregulation of MMP-9 indirectly affects T-cell polarization, and differentiated cytokine production, such as IL-12→Th1 or IL-4 and IL-5→Th2, increases the rate of basement membrane disruption (16). The interaction of T cells with mast cells activates and degranulates mast cells. Mast cell-derived TNF-α increases endothelial cell adhesion molecule expression and promotes lymphocyte adhesion and extravasation, and T cells secrete RANTES and MMP (17). RANTES results in sustained degranulation of mast cells, whereas MMP directly or indirectly disrupts the epithelial basement membrane (18). Thus, periodic interactions between T cells and mast cell secretion may be responsible for the long-term and chronic duration of the disease. IL-2, TNF-α and IFN-γ, representative cytokines of Th1 cells, may exacerbate disease severity and inhibit the differentiation of Th17 cells. At the same time, Th2 and Treg subsets may maintain the long-term chronicity of the disease by releasing IL-4 and TGF-β (19). Moreover, Th17 cells secreting IL-17, IL-21 and IL-22 may increase the inflammatory reaction. Furthermore, MMP derived from Th17 cells may damage the epithelial basement membrane, and Th9/IL-9 cells can exacerbate the occurrence of OLP disease by directly increasing Th17-cell levels or indirectly upregulating MMP9 levels in coordination with Th17 cells (20).

## 3 Functional cells and key chemokines that regulate the OLP immune microenvironment

Currently, since the etiology of OLP remains unknown, substantial evidence supports that T lymphocytes in OLP may be activated by presenting antigens through major histocompatibility (MHC) molecules and triggering keratinocyte apoptosis, suggesting that immune dysregulation may be of great importance in OLP pathogenesis (21). MMP overexpression and mast cell degranulation in OLP lesions further aggravate T-cell accumulation, basement membrane destruction and keratinocyte apoptosis (12, 22). These immune inflammatory cells and their cytokines in the immune microenvironment may affect OLP immune response characteristics, disease severity, and duration.

### 3.1 Functional cells regulate the OLP immune microenvironment

In OLP, there is strong crosstalk among keratinocytes, fibroblasts, macrophages, dendritic cells (DCs), mast cells, CD8^+^ T cells and Th cells (23). These interactions appear to continuously amplify and maintain the chronicity of inflammation. In OLP early stages, the proportions of Th cells, macrophages and DCs were higher than those in advanced stages, suggesting that in early stages, there is a predominance of antigen-presenting cells and T lymphocytes responsible for the induction of inflammatory responses, but CD8^+^ T cells present high levels in OLP advanced stages (24).

#### 3.1.1 Keratinocytes

Keratinocytes can produce the major constituent proteins that constitute the basement membrane to maintain their integrity and act as nondedicated antigen-presenting cells to identify, phagocytose, and present self-antigens. OLP is an inflammatory disease that affects the oral mucosa with the involvement of immune cells. At the cellular level, OLP may be due to immunologically induced degeneration of the basal layer (25). The target cell is epithelial basal cells in OLP, and the initial step may be antigen recognition through mucosal Langerhans cells. Basal keratinocytes present antigens with MHC class I molecules to CD8^+^ cytotoxic T cells and then induce them to produce a variety of inflammatory mediators that promote the liquefaction and degeneration of keratinocytes (26). Keratinocytes are not only target cells for immune response damage, but also intermediate mediators of the immune response involved in the immune response by releasing a series of cytokines. Studies have reported that IL-1β, tumor necrosis factor-α (TNF-α), GM-CSF, IL-8, RANTES, monocyte chemoattractant protein-1, and macrophage inflammatory protein-1 are elevated in OLP keratinocyte culture supernatants compared with the control group (27, 28). These cytokines enhance the expression of β2 integrin and ICAM-1, while upregulated adhesion molecules and cytokines may enhance monocyte migration (27–30). These results demonstrate that keratinocytes may be involved in the pathogenesis of OLP and that the inflammatory response at the lesion can be further exacerbated by the cytokines it produces.

#### 3.1.2 Fibroblasts

Fibroblasts are the main cellular components of connective tissue and have unique spindle structures (31). They not only function in the construction and remodeling of the extracellular matrix but are also key immune sentinel cells (32). Fibroblasts can detect damage-associated molecular patterns and pathogen-associated molecular patterns and activate proinflammatory signaling pathways to aid in leukocyte recruitment and regulate their activity; thus, these cells are now considered “nonclassical” branches of the innate immune system (33–36). In diseases such as chronic inflammatory diseases and cancer, inappropriate fibroblast activation promotes disease persistence by inducing proinflammatory and immunosuppressive properties, respectively (37). However, few studies have investigated the effects of OLP fibroblasts. Zhang Y et al. showed that the immune activation of OLP fibroblasts promotes CD4^+^ T-cell proliferation and migration and inhibits CD4^+^ T-cell apoptosis (38). Xu X et al. showed that fibroblasts located at OLP lesions upregulate IL-6, thus enhancing OLP angiogenesis and that anti-IL-6 receptor antibodies inhibit the OLP-interstitial IL-6 signaling pathway and inhibit OLP angiogenesis. This suggests that this may be a potential target for developing OLP therapies (39).

#### 3.1.3 Dendritic cells

Of note, DCs are dedicated antigen-presenting cells and are important components that induce the immunological response (40). CD4^+^ T helper and CD8^+^ cytotoxic T cells are activated when antigens are presented by MHC class I and class II molecules on dendritic cells, thereby inducing initial T-cell proliferation and initiating the immune response (41). Previous studies have shown increased aggregation of DC subsets in OLP lesion tissues, such as CD1a+/Langerin+ (Langerhans cells), DC-SIGN+ DCs, and CD123+/BDCA2+ plasmacyte-like DCs (PDCs) (26) (42). DCs in OLP can present antigens to CD4^+^ T cells, and their secreted interleukin-12 (IL-12) induces cytokine release from CD4^+^ T cells, including interferon gamma (IFN-γ) and IL-2, recruits CD8^+^ T cells to subepithelial regions and activates CD8^+^ T cells, eventually leading to the onset of chronic inflammation (43–45).

#### 3.1.4 Macrophages

Macrophages are differentiated from blood monocytes and are recruited to inflammatory sites by chemokines (46). Macrophages play a significant role in inflammation and cytotoxicity, chemokines (46). Macrophages play a significant role in pro-inflammation and cytotoxicity, phagocytosis of pathogens, immune surveillance, and immune regulation. Thus, they are the first line of defense in immune defense. Depending on their effector function, they can be classified as classically activated (M1) and alternatively activated (M2) macrophages (47). Infiltrating monocytes recruited to lesions differentiate into proinflammatory M1 macrophages at high levels of TNF-α, GM-CSF and IFN-γ, intensifying inflammation by the secretion of proinflammatory cytokines, including IL-1β and TNF-α, which upregulate cell adhesion molecules on the surface of endothelial cells and keratinocytes and induce T-cell chemokine expression (RANTES) to promote inflammatory cell recruitment (14–16, 23). Upregulation of matrix metalloproteinase 9 (MMP-9) indirectly affects the polarization of T cells and promotes basement membrane disruption by producing differentiated cytokines, such as IFN-γ and IL-12→Th1 or IL-4 and IL-2→Th2 (3, 48).

#### 3.1.5 Mast cells

Mast cells are derived from hematopoietic stem-fine, granular, mobile leukocytes with a wide range of functions, including inflammation, tissue repair, remodeling, and immune modulation (49). Compared with normal mucosal tissue, mast cells accumulated significantly more in OLP lesioned tissue and were mostly common in deeper connective tissue (50). The interaction of T cells with mast cells allows them to activate and degranulate mast cells, which secrete a series of cytokines and chemokines, such as chymase, tryptase, TNF-α and a range of interleukins, including IL-8 and IL-16 (51). Zhao et al. found that mast cell-derived TNF-α increases lymphocyte adhesion and extravasation by increasing the expression of endothelial cell adhesion molecules and promotes T-cell secretion of RANTES and MMP (17). RANTES results in sustained degranulation of mast cells, whereas MMP directly or indirectly disrupts the epithelial basement membrane (18). Thus, periodic interactions between T cells and mast cell secretion may be responsible for the long-term and chronic duration of the disease.

#### 3.1.6 T lymphocytes

The present study demonstrates that T lymphocyte-mediated liquefaction degeneration of basal keratinocytes leads to the occurrence of OLP, primarily in CD4^+^ T and CD8^+^ T cells. CD4^+^ T cells predominantly infiltrate connective tissue below the affected epithelium, namely, the subepithelial epithelium, whereas CD8^+^ T cells are usually located at the interface of epithelial and connective tissues, namely, the intraepithelial epithelium, sometimes adjacent to apoptotic keratinocytes (3, 52).

#### 3.1.7 CD4^+^ T

Currently, CD4^+^ T helper cells can be classified into Th1, Th2, Th17, Treg, Th9 and Tfh subsets based on the secretion of cytokines. IL-2, TNF-α and IFN-γ are representative cytokines of Th1 cells that activate macrophages and cytotoxic T lymphocytes, promote the production of cytokines, and participate in cellular immunity. Th2 cells promote B lymphocyte antibody production mainly through the secretion of cytokines such as IL-4 (53). The characteristic cytokine of Th1 cells is IFN-γ, which plays a key role in maintaining Th1 differentiation and proliferation, while IL-2 acts as a specific factor for Th2 cells and primarily promotes the clonal expansion of Th2 cells. The current measure of the Th1/Th2 balance is the ratio of IFN-γ/IL-4. IFN-γ and IL-4 were found to be significantly increased in OLP-diseased tissue, saliva and serum, suggesting that Th1 cells may be closely related to the development of OLP (54, 55). In addition, it has been reported that the levels of characteristic Th1 cytokines are lower in OLP patient serum than in normal control serum (56–58). Cytokines derived from Th1 cells inhibit the differentiation of Th17 cells (19). Our previous study showed that the reduced proportion of IFN-γ/IL-4 in OLP saliva suggests a possible dominance of Th2 cells in OLP saliva. Thus, increasing evidence suggests that Th1/Th2 imbalance may play a leading role in the occurrence of OLP (59). Th17 cells primarily secrete proinflammatory cytokines, including IL-17, IL-21 and IL-22 (60). Studies have shown that the level of IL-17 in serum as well as the proportion of Th17 cells in OLP lesion tissue and peripheral blood increased compared with healthy controls, suggesting that Th17 cells may be involved in the pathogenesis of OLP, especially in erosive OLP, whereas Th2 cells may be predominant in reticular OLP (20, 61). IL-23 was recently found to be a cytokine of the IL-12 family that maintains the differentiation and proliferation of Th17 cells (62–64). Lu et al. showed that both the IL-23p19 subunit and IL-17 are significantly elevated in OLP, demonstrating the selective regulatory role of the IL-23/IL-17 axis in OLP (65, 66).

The cell markers of Tregs are primarily CD25 and Foxp3, which maintain the body’s immune tolerance and homeostasis during the immune response by regulating the Th1/Th2 balance (67). Tao et al. found an increased proportion of CD4+CD25+ Treg+ cells in OLP lesion tissues and peripheral blood compared to normal controls (68). Treg cells mainly secrete specific cytokines, such as TGF-β, IL-10 and IL-35, of which TGF-β is the signature cytokine and can regulate the Th1/Th2 balance (69, 70). Furthermore, IL-10 secreted by Treg cells suppresses Th1 and Th2 cell proliferation (67). In addition, IL-35 can inhibit Th17 cells and Th1 cells through the proliferation of strong Tregs and IL-10 expression (71).

The Th22, Th9, and Tfh subsets are recently described subsets of CD4^+^ T cells. The former mainly secretes IL-22. Some studies have shown that IL-22 can promote OLP epithelial remodeling by inducing keratinocyte proliferation and epithelial cell proliferation and that TNF-α and IL-6 play crucial roles in the differentiation and proliferation of Th22 cells (72, 73). IL-9 is mainly produced by Th9 cells, and Wang et al. showed that Th9/IL-9 cells could exacerbate the occurrence of OLP by directly increasing Th17 cell levels or indirectly upregulating MMP9 levels in coordination with Th17 cells (20). The characteristic cytokine of the Tfh subset is IL-21, which promotes B-cell proliferation and autoantibody production and disrupts plasma cell differentiation (74). Tan et al. speculated that the Th1/Th2 imbalance might be partially attributed to the increase in Tfh-like cells (75).

#### 3.1.8 CD8^+^ T

CD8^+^ T cells play a vital role in the pathogenesis of OLP, especially in advanced OLP.

CD8^+^ T cells may migrate into the OLP epithelium through the basement membrane (BM) break region (21). It is mainly believed that CD8^+^ T lesion cells identify antigen-associated MHC class I on diseased keratinocytes and are activated by cytokines secreted by Th1 cells, which then cause keratinocyte apoptosis, leading to the occurrence of OLP (3). Possible mechanisms underlying apoptosis include (a) FAS-FAS ligand interactions between keratinocytes and cytotoxic T cells. (b) Cytotoxic T cells produce granzyme B in keratinocytes through perforin-induced membrane pores. (c) TNF-α secreted by cytotoxic T cells binds to the TNF-α receptor on the keratinocyte surface. Lage et al. confirmed the increased expression of granzyme B and perforin seen in OLP compared with cutaneous lichen planus and suggested that this increase is associated with the clinical behavior of the disease (13). These mechanisms activate a cysteine-containing aspartate proteollase (CaSpase)-mediated cascade, leading to apoptosis in keratinocytes.

### 3.2 Key secreted proteins regulate the OLP immune microenvironment

Cytokines are bioactive small peptide proteins that can be synthesized and secreted by various immune and nonimmune cells to regulate cell growth, function and differentiation and promote intercellular communication (76). They can induce each other to mediate local and systemic inflammation in an autocrine, paracrine, and endocrine manner (77). They generally initiate complex intercellular molecular interactions by binding the corresponding receptors, altering gene regulation, and resulting in altered phenotypic and functional changes in the target cells (78). Cytokine network imbalance may be related to immune-mediated OLP pathogenesis. In fact, overexpression of various proinflammatory factors, including interleukin (IL), IFN-γ and TNF-α, has been found in OLP lesions, peripheral blood, and saliva.

#### 3.2.1 Chemokines

Chemokines are proinflammatory cytokines that move cells direction toward high concentration stimuli and can induce directional chemotaxis in nearby response cells (79), which can be divided into four categories, CC, CXC, C and CX3C, according to their structure. RANTES, also known as CCL5, belongs to the CC chemokine family and could recruit lymphocytes, mast cells, monocytes, and natural killer cells in OLP (3). Shan et al. showed that CCL5 and CCR5 are upregulated in OLP, and the CCL5-CCR5 axis promotes CD4^+^ T lymphocyte aggregation in OLP tissues after inhibiting CCR5 expression while promoting T-cell apoptosis (80). This study also found that the pathological features of the oral mucosa in OLP patients can be formed by chemokines through regulating the migration of T lymphocytes, contributing to the emergence of T lymphocyte-infiltrating bands. Ichimura et al. showed that CXCL9, CXCL10, CXCL11, and CCL5 expression was higher in OLP than in normal tissues, which can recruit T lymphocytes, and the ligands of CXCR3 are CXCL9, CXCL10 and CXCL11, while the ligand of CCR5 is CCL5 (81). In addition, CXCR3 and CCR5 of Th1 cells are strongly expressed in OLP. This finding indicates that T-cell infiltration in OLP may be signaling through CXCR3 and CCR5.

#### 3.2.2 Interleukin

IL can be secreted by many kinds of cells and acts by transmitting information and immune regulation, and IL also plays a pivotal role in inflammatory processes in the human response. The major ILs currently are upregulated in OLP, such as IL-1, IL-2, IL-4, IL-5, IL-6, IL-8, IL-10, IL-12, IL-17 and IL-18 (82). IL-2 and its receptor IL-2R are highly expressed in OLP (65, 83). The IL-2/IL-2R signaling axis may regulate T-cell expansion and activation in foci (84). It was found that the B7-H1/PD-1 pathway negatively regulates IL-2, indicating that the IL-2/IL-2R signaling pathway may be a therapeutic target (85, 86). In addition to IL-2, IL-4 appears to be important in the pathogenesis of OLP because it can not only control Th2 cell differentiation but also negatively regulate Th1- and Th17-mediated inflammatory responses by inhibiting IL-17, IFN-γ and TNF-α production. Liu et al. found higher IL-4 levels in OLP saliva than in healthy controls (59), but studies also reported that there was no difference between OLP patients and normal controls (87). IL-6 is upregulated in OLP lesion tissues, saliva, and peripheral blood, especially in the eroded form, and may affect the infiltration of immunoinflammatory cells, leading to increased proinflammatory cytokine production in OLP lesions and exacerbating OLP inflammation (82, 88–90). All the above studies suggest an association between IL and the onset of OLP and that the proinflammatory effect of IL may aggravate the pathological injury of OLP. The detection of IL concentrations in OLP lesion tissue facilitates the clinical assessment of OLP patients.

#### 3.2.3 Tumor necrosis factor-α

TNF-α is an endotoxin-induced glycoprotein and is currently the most widely studied cytokine. Previous studies agreed that the expression of TNF-α was significantly elevated in both lesions and peripheral blood in OLP patients compared with healthy controls (3, 91, 92). Moreover, TNF-α was found to be overproduced in degranulated mast cells with OLP lesions (52). Topical steroids can inhibit elevated TNF-α in OLP lesions, indicating that TNF-α overexpression may play an essential role in OLP (93).

The study showed that TNF-α stimulation could induce RANTES, MMP-9 and TNF-α production and promote the expression of endothelial cell adhesion molecules in OLP, recruiting lymphocytes from blood vessels to extravascular regions (94–96). TNF-α can also contribute to epithelial basement membrane breakage by stimulating T-cell secretion and MMP activation (97). TNF-α also induces keratinocyte apoptosis by binding to TNF-α receptors on the surface of keratinocytes (91). In addition, it can cause secretion of RANTES in T cells to attract mast cells. TNF-α and thrombin released after degranulation can further stimulate more RANTES secretion, leading to chronic chemistry of OLP (52).

### 3.3 Other routes regulate the OLP immune microenvironment

Exosomes are extracellular vesicles with a diameter of 30-150 nm that can be secreted by T cells, B cells, hematopoietic cells, reticulocytes, dendritic cells, and tumor cells (98). Exosomes secreted by immune cells regulate the immune response and are associated with the pathogenesis of autoimmune diseases (99). Exosomes are newly recognized natural nanocarriers and intercellular messengers that have become important mediators of signal transmission. It has been suggested that aberrant expression of exosomal miRNAs may participate in the development of OLP (100, 101). Peng Q et al. found that circulating exosomal miR-34a-5p and miR-130b-3p were upregulated, while miR-301b-3p was downregulated in patients with OLP. Exosomal miR-34a-5p was positively correlated with the severity of OLP (102). In addition, they also found that in OLP, especially in erosive OLP, circulating exosomes significantly enhanced T-cell proliferation and migration, increased the proportion of IFN-γ/IL-4, and decreased apoptosis, which may accelerate the progression of OLP by regulating the T-cell-mediated inflammatory response (103). J-S Byun et al. reported that exosomal miR-4484 saliva was increased in OLP patients (104).

There are few articles investigating neutrophil extracellular traps (NETs) in OLP. They found that neutrophils isolated from the peripheral blood of OLP patients exhibit a potent ability to produce NETs, and the presence of excessive NETs may lead to many adverse consequences associated with OLP pathogenesis and the potential transition to OSCC (103). The inhibitory effect of flavonoids (epicatechin, catechin hydrate and rutin trihydrate, quercetin and luteolin) on NET formation in OLP patients (105, 106). Flavonoids can be considered a therapeutic strategy for NET-mediated diseases (such as OLP). However, the application of routes such as exosomes and NETs in the diagnosis and treatment of OLP still needs further study to design a correct therapeutic strategy for the disease.

## 4 Application potential of targeted immune microenvironment therapy for OLP

Since OLP is thought to be a T-cell-mediated disease associated with a Th1/Th2 imbalance in cytokine production, most therapeutic interventions are aimed at targeting the inflammatory pathway of OLP (44, 87, 107). Although steroids are the first-line medication for treating OLP, favorable evidence for their effectiveness is lacking. To date, multiple treatments have been used to treat symptomatic OLP, but it is difficult to completely cure due to its stubbornness. Thus, there is a tremendous challenge in treating symptomatic OLP, and there is now an urgent need to look for new therapies for symptomatic OLP.

### 4.1 Current immune-based therapies in OLP

Pharmacotherapy with corticosteroids, calcineurin inhibitors, thalidomide, mycophenolate mofetil (MMF), retinoids, BCG-PSN and traditional Chinese medicine (TCM) has been tried in clinical trials and has achieved varying degrees of success. The mode of action and effects of common drugs and drug categories of OLP are shown In **Table 1**, and the specific screening strategies were performed as described previously (126, 127). They can control the symptoms, but they are difficult to cure completely.

**Table 1:** Main mechanisms of action and efficacies of common drugs and drug categories used for treating oral lichen planus.

Drugs/drug class	Mechanism of action	Author, Year	Treatment scheme	Efficacy
Corticosteroids				
Triamcinolone	Reduces the exudation of leukocytes	Siponen et al, 2017 (108)	Group 1 (n = 11): tacrolimus 0.1% ointment, 3× daily for 3 weeks Group 2 (n = 7): triamcinolone acetonide 0.1%, 3× daily for 3 weeks Group 3 (n = 9): placebo (Orabase® paste), 3× daily for 3 weeks	Groups 1 and 2 showed a greater reduction in clinical score (p = 0.012 and 0.031, respectively) and VAS (44–77%) than placebo in the 3rd week, but with no significant difference between them. Groups 1 and 2 showed a 50% decrease in VAS after 6 months of treatment
		Singh et al, 2017 (109)	Group 1 (n = 10): 0.1% triamcinolone acetonide 0.1%, 2× daily for 3 months Group 2 (n = 10): oral dapsone 100 mg + iron and folic acid tablet, 2× daily for 3 months Group 3 (n = 10): tacrolimus 0.1%, 2× daily for 3 months Group 4 (n = 10): retinoid, 2× daily for 3 months	The four treatment groups showed significant clinical improvement in signs and symptoms after 3 months (p < 0.05), with no difference between them. Among nonsteroidal drugs, oral dapsone showed a better response than topical retinoid (p < 0.05).
		Sivaraman et al, 2016 (110)	Group 1 (n = 10): triamcinolone acetonide 0.1%, 3× daily for 6 weeks Group 2 (n = 10): clobetasol propionate 0.05%, 3× daily for 6 weeks Group 3 (n = 10): tacrolimus 0.03%, 3× daily for 6 weeks	At the end of treatment, there was a significant difference between the 3 groups (p = 0.004), with group 2 being better than group 3 (p = 0.007). There was no recurrence after 3 months of follow-up.
		Vohra, Singal, Sharma, 2016 (111)	Group 1 (n = 20): tacrolimus 0.1% ointment, 2× daily for 8 weeks Group 2 (n = 20): pimecrolimus 1%, 2× daily for 8 weeks	Both groups showed a statistically significant reduction in clinical score and mean serum levels of IL-6 and IL-8 at the end of treatment and follow-up compared to baseline (p < 0.05), but without significant differences between them.
Clobetasol	Inhibits the lysozyme release and phagocytosis	Hettiarachchi et al, 2017 (112)	Group 1 (n = 34): clobetasol propionate 0.05% cream, 2× daily + nystatin mouthwash, 2× daily for 3 weeks Group 2 (n = 34): tacrolimus (0.1%), 2× daily + nystatin mouthwash, 2× daily for 3 weeks	Statistically significant reduction in mean VAS and clinical score between end of treatment (3 weeks) and after 5 weeks compared to baseline in groups 1 and 2 (p < 0.05).
		Sivaraman et al, 2016 (110)	Group 1 (n = 10): triamcinolone acetonide 0.1%, 3× daily for 6 weeks Group 2 (n = 10): clobetasol propionate 0.05%, 3× daily for 6 weeks Group 3 (n = 10): tacrolimus 0.03%, 3× daily for 6 weeks	At the end of treatment, there was a significant difference between the 3 groups (p = 0.004), with group 2 being better than group 3 (p = 0.007). There was no recurrence after 3 months of follow-up.
Betamethasone	Inhibits the proliferation of fibroblasts	Samimi et al, 2020 (113)	Group 1 (n = 36): betamethasone dipropionate ointment 0.05% + oral placebo solution (excipient of solution, Phosal), 2× daily for 3 months Group 2 (n = 39): rapamycin oral solution 1 mg/ml + placebo (Vaseline ointment), 2× daily for 3 months	There was no difference between groups when compared to clinical remission. However, betamethasone showed a significantly greater reduction in VAS compared to rapamycin at the end of 3 months. At the end of the follow-up, there was no difference between the groups.
		Ezzatt, Helmy, 2018 (114)	Group 1 (n = 15): pimecrolimus 1% cream, 0.5 ml 4× daily for 4 weeks Group 2 (n = 15): betamethasone 17-valerate 0.1%, 0.5 ml 4× daily for 4 weeks	Groups 1 and 2 showed a reduction in clinical score (p < 0.001), VAS (p < 0.001), and expression of CD133 after 4 weeks of treatment compared to baseline (p < 0.002), and this reduction was significantly smaller in group 1.
Fluocinonide – 0.1%、0.05%	Inhibits the pro-inflammatory cytokines IL-1, IL-2, IL-3, IL-6, TNF-α, GM-CSF, IFN-γ	Carbone et al, 1999 (115)	Group 1 (n = 25): 0.05% clobetasol propionate ointment, 2× daily for 4 months, 1× daily for 2 months Group 2 (n = 24): 0.05% fluocinonide ointment, 3× daily for 2 months, 2× daily for 2 months, 1× daily for 2 months Group 3 (n = 11): hydroxyethyl cellulose gel and antimycotic, 2× daily for 6 months	All patients treated with clobetasol and 90% of the patients treated with fluocinonide witnessed some improvement, whereas in the placebo group only 20% of patients improved (P < 0.0001 and P= 0.00029, respectively)
Calcineurin inhibitors				
tacrolimus	inhibit cytokine production and T-cell activation, reduce Treg proliferation and downregulate NF-κB signaling, suppresses mast cell activation and affects Langerhans cell function	Singh et al, 2017 (109)	Group 1 (n = 10): 0.1% triamcinolone acetonide 0.1%, 2× daily for 3 months Group 2 (n = 10): oral dapsone 100 mg + iron and folic acid tablet, 2× daily for 3 months Group 3 (n = 10): tacrolimus 0.1%, 2× daily for 3 months Group 4 (n = 10): topical retinoid, 2× daily for 3 months	The four treatment groups showed significant clinical improvement in signs and symptoms after 3 months (p < 0.05), with no difference between them. Among nonsteroidal drugs, oral dapsone had a lower score of signs and symptoms than topical retinoid (p < 0.05).
		Hettiarachchi et al, 2017 (112)	Group 1 (n = 34): clobetasol propionate 0.05% cream, 2× daily + nystatin mouthwash, 2× daily for 3 weeks Group 2 (n = 34): tacrolimus (0.1%), 2× daily + nystatin mouthwash, 2× daily for 3 weeks	Statistically significant reduction in mean VAS and clinical score between end of treatment (3 weeks) and after 5 weeks compared to baseline in groups 1 and 2 (p < 0.05).
		Siponen et al, 2017 (108)	Group 1 (n = 11): tacrolimus 0.1% ointment, 3× daily for 3 weeks Group 2 (n = 7): triamcinolone acetonide 0.1%, 3× daily for 3 weeks Group 3 (n = 9): placebo (Orabase® paste), 3× daily for 3 weeks	Groups 1 and 2 showed a greater reduction in clinical score (p = 0.012 and 0.031, respectively) and VAS (44–77%) than placebo in the 3rd week, but with no significant difference between them. Groups 1 and 2 showed a 50% decrease in VAS after 6 months of treatment
		Sivaraman et al, 2016 (110)	Group 1 (n = 10): triamcinolone acetonide 0.1%, 3× daily for 6 weeks Group 2 (n = 10): clobetasol propionate 0.05%, 3× daily for 6 weeks Group 3 (n = 10): tacrolimus 0.03%, 3× daily for 6 weeks	At the end of treatment, there was a significant difference between the 3 groups (p = 0.004), with group 2 being better than group 3 (p = 0.007). There was no recurrence after 3 months of follow-up.
		Vohra, Singal, Sharma, 2016 (111)	Group 1 (n = 20): tacrolimus 0.1% ointment, 2× daily for 8 weeks Group 2 (n = 20): pimecrolimus 1%, 2× daily for 8 weeks	Both groups showed a statistically significant reduction in clinical score and mean serum levels of IL-6 and IL-8 at the end of treatment and follow-up compared to baseline (p < 0.05), but without significant differences between them.
pimecrolimus	reduces cytokine and CD95 (Fas) expression, inhibit T-cell activation	Ezzatt, Helmy, 2018 (116)	Group 1 (n = 15): pimecrolimus 1% cream, 0.5 ml 4× daily for 4 weeks Group 2 (n = 15): betamethasone 17-valerate 0.1%, 0.5 ml 4× daily for 4 weeks	Groups 1 and 2 showed a reduction in clinical score (p < 0.001), VAS (p < 0.001), and expression of CD133 after 4 weeks of treatment compared to baseline (p < 0.002), and this reduction was significantly smaller in group 1.
		Riaz et al, 2017 (117)	Group 1 (n = 18): pimecrolimus 1% cream, 4× daily for 8 weeks Group 2 (n = 18): Triamcinolone acetonide 0.1% paste 3× daily for 8 weeks	There was a significantly greater reduction in VAS in group 1 at 4 and 8 weeks, as well as at follow-up.
		Vohra, Singal, Sharma, 2016 (111)	Group 1 (n = 20): tacrolimus 0.1% ointment, 2× daily for 8 weeks Group 2 (n = 20): pimecrolimus 1%, 2× daily for 8 weeks	Groups 1 and 2 showed a statistically significant reduction in clinical score and mean serum levels of IL-6 and IL-8 at the end of treatment and follow-up compared to baseline (p < 0.05), but without significant differences between groups.
cyclosporine	inhibit NF-κB signaling, the immortalized proliferation of keratinocytes, and Treg proliferation and activation	Thongprasom et al, 2007 (118)	Group 1 (n = 7): triamcinolone acetonide 0.1%, orabase, 3× daily for 8 weeks Group 2 (n = 6): cyclosporine 100 mg/ml solution, mouthwash 3× daily for 8 weeks	There was no significant difference between the groups regarding the clinical response and the burning sensation (p > 0.01). At week 8, 50% of group participants had a complete clinical response and 50% partial response. In group 2, 33.33% had a partial clinical response and 66.66% had no response.
		Yoke et al, 2006 (119)	Group 1 (n = 71): triamcinolone acetonide 0.1%, orabase, 3× daily for 8 weeks Group 2 (n = 68): cyclosporine 100 mg/ml solution, mouthwash 3× daily for 8 weeks	There were no significant differences between groups at 4 and 8 weeks.
		Conrotto et al, 2006 (120)	Group 1 (n = 19): clobetasol propionate gel 0.025%, 2× daily for 8 weeks + miconazole gel 1× daily + chlorhexidine 0.12% mouthwash, 3× daily Group 2 (n = 20): cyclosporine gel 1.5%; 2× daily for 8 weeks + miconazole gel 1× daily + chlorhexidine 0.12% mouthwash, 3× daily	After 4 weeks, group 1 had a greater number of patients (94.73%) with complete or partial response, when compared to group 2 (65%) (p = 0.04). There was no difference between groups regarding reduction of symptoms. In group 2, there was greater stability of the lesions after 2 months of follow-up (p = 0.04).
Monoclonal antibodies				
Efalizumab	block T-cell activation and migration	Heffernan et al. 2007 (121)	Group 1 (n=4): 0.7 mg/kg subcutaneously at week 0 followed by 1.0 mg/kg weekly from week 1 to week 11	The mean reduction in the affected mucosal surface area was 71.1% (range 57.3% to 96.8%). The mean improvement in the 100-mm VAS for pain was 82%.
Others				
Shalidomide	suppress Th-cell activity and NF-κB signaling, reduce the production of cytokines such as TNF-α, IFN-γ, VEGF, and IL-6	Wu et al, 2010 (122)	Group 1 (n = 37): thalidomide 1% paste, 3× daily for 1 week Group 2 (n = 32): dexamethasone 0.043% paste, 3× daily for 1 week Patients who did not obtain a complete response, continued treatment 3× daily per week for an additional 3 weeks	After 1 week of application, both groups showed significant reductions in erosive areas and VAS scores (*p* < 0.001), but with no difference between them. Regarding the recurrence of injuries, there was also no statistically significant difference between the groups.
Mycophenolate mofetil (MMF)	suppresses dendritic cell (DC) maturation and antibody formation, and inhibit the proliferation of activated T cells	Samiee et al, 2020 (123)	Group A (n = 10): bilateral oral lichen planus. Mycophenolate mofetil 2% mucoadhesive on the lesion on one side, 2× daily for 4 weeks. Placebo applied on the lesion on the other side, 2× a day, for 4 weeks Group B (n = 17): unilateral oral lichen planus. Mycophenolate mofetil 2%, 2× for 4 weeks	There was no difference between the size of the lesions that received the immunomodulator in relation to the placebo. At the end of 4 weeks of treatment, the group that received mycophenolate mofetil reduced the size of the lesion compared to baseline. While the placebo reduced pain and burning after 4 weeks when compared to its baseline.
BCG-PSN	modulates T-cell subsets, elevates the secretion of IFN-γ and regulates the imbalanced state of the IFN-γ/IL-4 cytokine ratio	Mohamad et al. 2017 (124)	n=11: 0.5 ml BCG-PSN twice weekly for three weeks	9 of 11 BCG-PSN-treated patients showed complete response
Retinoids	regulation function on T cells and macrophages	Xiong et al, 2009 (125)	Group 1 (n = 31): 0.5 ml BCG-PSN every other day for 2 weeks Group 2 (n = 25): 10 mg triamcinolone acetonide, every week for 2 weeks	27 of 31 BCG-PSN-treated patients (87.1%) and 22 of 25 TA-treated patients (88.0%) healed. There were no significant differences between the two groups in erosive areas (27.86 +/- 27.97 vs. 25.68 +/- 34.65, P = 0.801) and VAS scores (2.45 +/- 1.64 vs. 2.40 +/- 1.38, P = 0.946).

Visual analog scale/score: VAS

Topical corticosteroids can serve as first-line medicine to treat OLP, and medications for topical treatment include triamcinolone acetonide, clobetasol propionate, fluocinonide, fluocinolone acetonide, fluticasone propionate, and betamethasone pentate/sodium phosphate. Systemic corticosteroids are suitable for severe, extensive, or erosive OLP or drug-resistant/refractory OLP (113–116). It primarily inhibits the CD4^+^ Th subpopulation of OLP patients. For instance, 0.1% fluocinolone acetonide has an immunomodulatory effect on the reduction of IFN-γ (128).

Calcineurin inhibitors mainly include tacrolimus, pimecrolimus, and cyclosporine, second-line medicines to treat OLP. Calcineurin inhibitors primarily inhibit IL-2 transcription, reducing T-cell activation (111, 129, 130). Tacrolimus inhibits cytokine production and T-cell activation, reduces Treg proliferation, and downregulates NF-κB signaling (112). In addition, tacrolimus suppresses mast cell activation and affects Langerhans cell function (68, 108, 110, 118, 131). However, due to symptoms such as burning sensation occurring for a short time and relapses of OLP after cessation, it is advocated for short-term use (132, 133). Pimecrolimus first reduces cytokine synthesis and release to inhibit T-cell activation and then reduces CD95 (Fas) expression on keratinocytes in OLP (117, 134). Cyclosporine can induce T-cell apoptosis by inhibiting NF-κB signaling, inhibiting the immortalized proliferation of keratinocytes, and inhibiting Treg proliferation and activation (119, 120, 135, 136). Tacrolimus and pimecrolimus have a higher safety profile than cyclosporine (116). Thalidomide has antiangiogenic, anti-inflammatory, and anti-immune properties that suppress Th-cell activity, inhibit the production of TNF-α by promoting TNF-α mRNA degradation and inhibiting NF-κB activity and reduce IFN-γ, VEGF, and IL-6 production (122, 137). Mycophenolate mofetil (MMF) is an immunosuppressor that suppresses DC maturation and antibody formation and inhibits activated T-cell proliferation in a reversible and specific way. However, it is not recommended because there is not sufficient evidence to support OLP treatment (123, 138, 139). Retinoids also have a regulatory function on T cells and macrophages (109, 124, 140, 141).

Efalizumab is a recombinant humanized monoclonal IgG1 antibody against CD11a, which is a subunit of LFA-1 (lymphocyte function-associated antigen 1) (142). Efalizumab can reduce T lymphocyte infiltration and activation by binding to CD11a, thereby blocking the interactions between LFA-1, which is expressed on the surface of endothelial cells and T lymphocytes, and ICAM-1, which is expressed on antigen-presenting cells (143). It has been shown that Efalizumab may be useful in the treatment of erosive oral lichen planus (121).

BCG-PSN, a novel potent bioimmunomodulator, modulates T-cell subsets and maintains the proportion of CD4^+^ T/CD8^+^ T cells at normal levels; furthermore, it also elevates the secretion of IFN-γ and regulates the imbalanced state of the IFN-γ/IL-4 cytokine ratio (144, 145). In addition, a previous *in vitro* study found that BCG-PSN could upregulate IFN-γ secretion from the PBMCs of OLP patients, increase the ratio of IFN-γ/IL-4 cytokines, and regulate Th1/Th2 disequilibrium in OLP (146). Additionally, BCG-PSN can serve as an alternative treatment for erosive OLP (125, 145).

Traditional Chinese medicine (TCM) regulates the function of internal organs based on the overall concept and syndrome differentiation. The treatment of OLP in TCM can better avoid the adverse reactions caused by the long-term use of Western medicine and the problems that easily relapse after drug withdrawal (147). The first two types of TCM to treat oral lichen planus are heat-clearing and tonifying deficiency tonic drugs, followed by wet medicine, promoting blood circulation and removing blood stasis drugs (148). T lymphocyte function is diminished, and T-cell subsets are unbalanced in OLP patients (149). TCM treatment has a two-way regulatory effect on patients’ immunity, which can restore the immune balance and achieve good clinical results (150).

### 4.2 Therapeutic prospects for OLP based on the immune microenvironment

**Figure 2:**
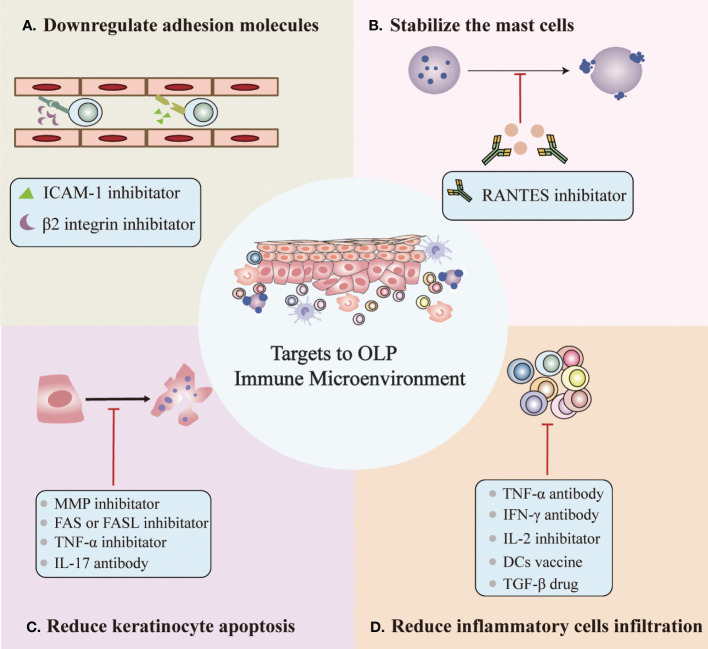
Potential targets for OLP based on the immune microenvironment. **(A)** Downregulation of the expression of surface adhesion molecules on endothelial cells and keratinocytes. **(B)** Stabilization of mast cells to prevent degranulation. **(C)** Reduced keratinocyte apoptosis. **(D)** Reduced inflammatory cell infiltration.

In addition to known therapeutic targets, we propose potential therapeutic targets based on the OLP immune microenvironment, which can be roughly divided into four categories (**Figure 2**) (1): Downregulation of the expression of surface adhesion molecules on endothelial cells and keratinocytes, such as β2 integrin and ICAM-1 inhibitors (27, 30) (2). Stabilize mast cells to prevent degranulation, such as the RANTES inhibitor mast (3, 17, 18, 51) (3). Reduced keratinocyte apoptosis, e.g., MMP inhibitor, FAS or FASL inhibitor, TNF-α or TNF-αR inhibitor and IL-17 antibody (13, 91) (4). Reduced inflammatory cell infiltration, such as TNF-α antibody, IFN-γ antibody, IL-2 antibody, DC vaccine, and TGF-β drug (27, 44, 45, 52). However, potential therapeutic targets based on the OLP immune microenvironment require additional research before they can be clinically applied.

## 5 Conclusions and further perspectives

In recent years, although significant progress has been made in the research of the OLP immune microenvironment, there are still many aspects worth our deep investigation, especially the intrinsic mechanisms by which the immune components in the immune microenvironment led to the chronic inflammatory environment of OLP, highlighting the need for further innovation in the field. Moreover, the antigenic nature of the initial events that induce OLP pathogenesis remains elusive. However, it is becoming increasingly clear that immune components in the immune microenvironment play an extremely key role in the pathogenesis of OLP, with numerous factors working in concert to form unique immune modulatory modes in the OLP immune microenvironment. Furthermore, studies of the OLP immune microenvironment will facilitate further exploration of therapeutic strategies against OLP, indicating new directions for developing safer and more effective therapeutic targets. Although there are several targeted immune microenvironment therapies or treatments, new alternative therapies are highly appreciated due to the lack of any strong evidence for OLP treatment. As we mentioned above, blocking either IL-2, IL-17, TNF-α, IFN-γ, MMP-9 or RANTES activity or upregulating TGF-β activity in OLP may have therapeutic value. To date, our study of OLP remains at the cellular level, and there is no appropriate animal model for clinical patients with OLP. Therefore, more studies are needed to obtain accurate animal models of OLP, so the efficacy observed by blocking or upregulating one factor or multiple factors can be verified at the animal level. In the future, we can adopt emerging technologies such as mass spectrometry flow cytometry and single-cell RNA sequencing to explore the immune cell subtypes and discover new cell subtypes in the OLP immune microenvironment and through a multiplex immunohistochemistry approach to explore the exact role of immune cells and cytokines and their differential changes in the process of treatment to further explore the interaction relationship among immune inflammatory cells, keratinocytes and cytokines and how they can synergize or antagonize each other to affect ongoing inflammation in the OLP immune microenvironment.

## Author contributions

JL and LJ contributed to designing the manuscript. XD, YW and QC drafted the manuscript. All authors contributed to the article and approved the submitted version.

## References

[1] Hamour AF Klieb H Eskander A. Oral lichen planus. CMAJ (2020) 192(31):E892. doi: 10.1503/cmaj.200309 32753462 PMC7828879

[2] Warnakulasuriya S Kujan O Aguirre-Urizar JM Bagan JV González-Moles SMÁ Kerr AR. Oral potentially malignant disorders: A consensus report from an international seminar on nomenclature and classification, convened by the WHO collaborating centre for oral cancer. Oral Dis (2021) 27(8):1862–80. doi: 10.1111/odi.13704 33128420

[3] Sugerman PB Savage NW Walsh LJ Zhao ZZ Zhou XJ Khan A. The pathogenesis of oral lichen planus. Crit Rev Oral Biol Med (2002) 13(4):350–65. doi: 10.1177/154411130201300405 12191961

[4] Triolo Va. Nineteenth century foundations of cancer research advances in tumour pathology, nomenclature, and theories of oncogenesis. Cancer Res (1965) 25:75–106. 14264062

[5] O'Byrne KJ Dalgleish AG. Chronic immune activation and inflammation as the cause of malignancy. Br J Cancer (2001) 85(4):473–83. doi: 10.1054/bjoc.2001.1943 PMC2364095 11506482

[6] Weitzman SA Gordon LI. Inflammation and cancer: role of phagocyte-generated oxidants in carcinogenesis. Blood (1990) 76(4):655–63. doi: 10.1182/blood.V76.4.655.655 2200535

[7] Balkwill F Mantovani A. Inflammation and cancer: back to virchow? Lancet (2001) 357(9255):539–45. doi: 10.1016/S0140-6736(00)04046-0 11229684

[8] Pupa SM Ménard S Forti S Tagliabue E. New insights into the role of extracellular matrix during tumor onset and progression. J Cell Physiol (2002) 192(3):259–67. doi: 10.1002/jcp.10142 12124771

[9] Mignogna MD Fedele S Lo Russo L Lo Muzio L Bucci E. Immune activation and chronic inflammation as the cause of malignancy in oral lichen planus: is there any evidence? Oral Oncol (2004) 40(2):120–30. doi: 10.1016/j.oraloncology.2003.08.001 14693234

[10] Farlow JL Brenner JC Lei YL Chinn SB. Immune deserts in head and neck squamous cell carcinoma: A review of challenges and opportunities for modulating the tumor immune microenvironment. Oral Oncol (2021) 120:105420. doi: 10.1016/j.oraloncology.2021.105420 34218062 PMC8753751

[11] Roopashree MR Gondhalekar RV Shashikanth MC George J Thippeswamy SH Shukla A. Pathogenesis of oral lichen planus–a review. J Oral Pathol Med (2010) 39(10):729–34. doi: 10.1111/j.1600-0714.2010.00946.x 20923445

[12] Sugerman PB Satterwhite K Bigby M. Autocytotoxic T-cell clones in lichen planus. Br J Dermatol (2000) 142(3):449–56. doi: 10.1046/j.1365-2133.2000.03355.x 10735949

[13] Lage D Pimentel VN Soares TC Souza EM Metze K Cintra ML. Perforin and granzyme b expression in oral and cutaneous lichen planus - a comparative study. J Cutan Pathol (2011) 38(12):973–8. doi: 10.1111/j.1600-0560.2011.01781.x 22050094

[14] Sugermann PB Savage NW Seymour GJ Walsh LJ. Is there a role for tumor necrosis factor-alpha (TNF-alpha) in oral lichen planus? J Oral Pathol Med (1996) 25(5):219–24. doi: 10.1111/j.1600-0714.1996.tb01375.x 8835818

[15] Li J Farthing PM Thornhill MH. Oral and skin keratinocytes are stimulated to secrete monocyte chemoattractant protein-1 by tumour necrosis factor-alpha and interferon-gamma. J Oral Pathol Med (2000) 29(9):438–44. doi: 10.1034/j.1600-0714.2000.290904.x 11016686

[16] Ferrisse TM de Oliveira AB Palaçon MP Silva EV Massucato EMS de Almeida LY. The role of CD68+ and CD163+ macrophages in immunopathogenesis of oral lichen planus and oral lichenoid lesions. Immunobiology (2021) 226(3):152072. doi: 10.1016/j.imbio.2021.152072 33677150

[17] Zhao ZZ Savage NW Sugerman PB Walsh LJ. Mast cell/T cell interactions in oral lichen planus. J Oral Pathol Med (2002) 31(4):189–95. doi: 10.1034/j.1600-0714.2002.310401.x 12076321

[18] Ramalingam S Malathi N Thamizhchelvan H Sangeetha N Rajan ST. Role of mast cells in oral lichen planus and oral lichenoid reactions. Autoimmune Dis (2018) 2018:7936564. doi: 10.1155/2018/7936564 29593898 PMC5822832

[19] Mucida D Salek-Ardakani S. Regulation of TH17 cells in the mucosal surfaces. J Allergy Clin Immunol (2009) 123(5):997–1003. doi: 10.1016/j.jaci.2009.03.016 19362732 PMC2679861

[20] Wang H Bai J Luo Z Fu J Wang H Sun Z. Overexpression and varied clinical significance of Th9 versus Th17 cells in distinct subtypes of oral lichen planus. Arch Oral Biol (2017) 80:110–6. doi: 10.1016/j.archoralbio.2017.04.003 28412610

[21] Zhou XJ Sugerman PB Savage NW Walsh LJ Seymour GJ. Intra-epithelial CD8+ T cells and basement membrane disruption in oral lichen planus. J Oral Pathol Med (2002) 31(1):23–7. doi: 10.1046/j.0904-2512.2001.10063.x 11896819

[22] Srinivas K Aravinda K Ratnakar P Nigam N Gupta S. Oral lichen planus - review on etiopathogenesis. Natl J Maxillofac Surg (2011) 2(1):15–6. doi: 10.4103/0975-5950.85847 PMC3304232 22442603

[23] Payeras MR Cherubini K Figueiredo MA Salum FG. Oral lichen planus: focus on etiopathogenesis. Arch Oral Biol (2013) 58(9):1057–69. doi: 10.1016/j.archoralbio.2013.04.004 23660124

[24] De Panfilis G Manara GC Allegra F. Remarks on early versus late lichen planus. Arch Dermatol Res (1981) 270(2):163–6. doi: 10.1007/BF00408227 6972737

[25] Sugerman PB Savage NW. Oral lichen planus: causes, diagnosis and management. Aust Dent J (2002) 47(4):290–7. doi: 10.1111/j.1834-7819.2002.tb00540.x 12587763

[26] Kumar TA Veeravarmal V Nirmal RM Amsaveni R Nassar MHM Kesavan G. Expression of cluster of differentiation 1a-positive langerhans cells in oral lichen planus. Indian J Dermatol (2019) 64(1):41–6. doi: 10.4103/ijd.IJD_350_16 PMC6340233 30745634

[27] Yamamoto T Nakane T Osaki T. The mechanism of mononuclear cell infiltration in oral lichen planus: the role of cytokines released from keratinocytes. J Clin Immunol (2000) 20(4):294–305. doi: 10.1023/a:1006671804110 10939717

[28] Bacon KB Westwick J Camp RD. Potent and specific inhibition of IL-8-, IL-1 alpha- and IL-1 beta-induced *in vitro* human lymphocyte migration by calcium channel antagonists. Biochem Biophys Res Commun (1989) 165(1):349–54. doi: 10.1016/0006-291x(89)91076-0 2686646

[29] Wang JM Colella S Allavena P Mantovani A. Chemotactic activity of human recombinant granulocyte-macrophage colony-stimulating factor. Immunology (1987) 60(3):439–44. doi: 10.1016/0006-291x(89)91076-0 PMC1453246 3494669

[30] Little MC Griffiths CE Watson RE Pemberton MN Thornhill MH. Oral mucosal keratinocytes express RANTES and ICAM-1, but not interleukin-8, in oral lichen planus and oral lichenoid reactions induced by amalgam fillings. Clin Exp Dermatol (2003) 28(1):64–9. doi: 10.1046/j.1365-2230.2003.01158.x 12558634

[31] Molenaar JC. Uit de bibliotheek van het nederlands tijdschrift voor geneeskunde. Rudolf virchow: die cellularpathologie in ihrer begründung auf physiologische und pathologische gewebelehre; 1858 [From the library of the Netherlands journal of medicine. Rudolf virchow: Die cellularpathologie in ihrer begründung auf physiologische und pathologische gewebelehre; 1858]. Ned Tijdschr Geneeskd (2003) 147(45):2236–44. doi: 10.1046/j.1365-2230.2003.01158.x 14640063

[32] Tarin D Croft CB. Ultrastructural features of wound healing in mouse skin. J Anat (1969) 105(Pt 1):189–90. 5803198

[33] Pierer M Rethage J Seibl R Lauener R Brentano F Wagner U. Chemokine secretion of rheumatoid arthritis synovial fibroblasts stimulated by toll-like receptor 2 ligands. J Immunol (2004) 172(2):1256–65. doi: 10.4049/jimmunol.172.2.1256 14707104

[34] Bombardieri M Kam NW Brentano F Choi K Filer A Kyburz D. A BAFF/APRIL-dependent TLR3-stimulated pathway enhances the capacity of rheumatoid synovial fibroblasts to induce AID expression and ig class-switching in b cells. Ann Rheum Dis (2011) 70(10):1857–65. doi: 10.1136/ard.2011.150219 21798884

[35] Brentano F Schorr O Gay RE Gay S Kyburz D. RNA Released from necrotic synovial fluid cells activates rheumatoid arthritis synovial fibroblasts *via* toll-like receptor 3. Arthritis Rheum (2005) 52(9):2656–65. doi: 10.1002/art.21273 16142732

[36] Seki E Brenner DA. Toll-like receptors and adaptor molecules in liver disease: update. Hepatology (2008) 48(1):322–35. doi: 10.1002/hep.22306 18506843

[37] Davidson S Coles M Thomas T Kollias G Ludewig B Turley S. Fibroblasts as immune regulators in infection, inflammation and cancer. Nat Rev Immunol (2021) 21(11):704–17. doi: 10.1038/s41577-021-00540-z 33911232

[38] Zhang Y Liu K Cheng J Zhou C Zhang M Fan Y. FAP-α+ immunofibroblasts in oral lichen planus promote CD4+ T-cell infiltration *via* CCL5 secretion. Exp Dermatol (2022) 31(9):1421–30. doi: 10.1111/exd.14613 35598279

[39] Xu XH Liu Y Feng L Yang YS Liu SG Guo W. Interleukin-6 released by oral lichen planus myofibroblasts promotes angiogenesis. Exp Ther Med (2021) 21(4):291. doi: 10.3892/etm.2021.9722 33717234 PMC7885057

[40] Banchereau J Steinman RM. Dendritic cells and the control of immunity. Nature (1998) 392(6673):245–52. doi: 10.1038/32588 9521319

[41] Banchereau J Briere F Caux C Davoust J Lebecque S Liu YJ. Immunobiology of dendritic cells. Annu Rev Immunol (2000) 18:767–811. doi: 10.1146/annurev.immunol.18.1.767 10837075

[42] Santoro A Majorana A Roversi L Gentili F Marrelli S Vermi W. Recruitment of dendritic cells in oral lichen planus. J Pathol (2005) 205(4):426–34. doi: 10.1002/path.1699 15714455

[43] Kang K Kubin M Cooper KD Lessin SR Trinchieri G Rook AH. IL-12 synthesis by human langerhans cells. J Immunol (1996) 156(4):1402–7. doi: 10.1002/path.1699 8568240

[44] Simark Mattsson C Jontell M Bergenholtz G Heyden M Dahlgren UI. Distribution of interferon-gamma mRNA-positive cells in oral lichen planus lesions. J Oral Pathol Med (1998) 27(10):483–8. doi: 10.1111/j.1600-0714.1998.tb01917.x 9831961

[45] Farthing PM Cruchley AT. Expression of MHC class II antigens (HLA DR, DP and DQ) by keratinocytes in oral lichen planus. J Oral Pathol Med (1989) 18(5):305–9. doi: 10.1111/j.1600-0714.1989.tb00402.x 2475620

[46] Merry R Belfield L McArdle P McLennan A Crean S Foey A. Oral health and pathology: a macrophage account. Br J Oral Maxillofac Surg (2012) 50(1):2–7. doi: 10.1016/j.bjoms.2010.10.020 21310515

[47] Gordon S Martinez FO. Alternative activation of macrophages: mechanism and functions. Immunity (2010) 32(5):593–604. doi: 10.1016/j.immuni.2010.05.007 20510870

[48] Mosmann TR Cherwinski H Bond MW Giedlin MA Coffman RL. Two types of murine helper T cell clone. i. definition according to profiles of lymphokine activities and secreted proteins. J Immunol (1986) 136(7):2348–57. doi: 10.1016/j.immuni.2010.05.007 2419430

[49] Walsh LJ. Mast cells and oral inflammation. Crit Rev Oral Biol Med (2003) 14(3):188–98. doi: 10.1177/154411130301400304 12799322

[50] Sharma R Sircar K Singh S Rastogi VX. Role of mast cells in pathogenesis of oral lichen planus. J Oral Maxillofac Pathol (1991) 15(3):267–71. doi: 10.4103/0973-029X.86674 PMC3227251 22144827

[51] Galli SJ Gordon JR Wershil BK. Cytokine production by mast cells and basophils. Curr Opin Immunol (1991) 3(6):865–72. doi: 10.1016/s0952-7915(05)80005-6 1793528

[52] Zhao ZZ Sugerman PB Zhou XJ Walsh LJ Savage NW. Mast cell degranulation and the role of T cell RANTES in oral lichen planus. Oral Dis (2001) 7(4):246–51. doi: 10.1034/j.1601-0825.2001.70408.x 11575876

[53] Wang L Wu W Chen J Li Y Xu M Cai Y. MicroRNA microarray-based identification of involvement of miR-155 and miR-19a in development of oral lichen planus (OLP) by modulating Th1/Th2 balance *via* targeting eNOS and toll-like receptor 2 (TLR2). Med Sci Monit (2018) 24:3591–603. doi: 10.12659/MSM.907497 PMC6003260 29813046

[54] Wang Y Zhou J Fu S Wang C Zhou B. A study of association between oral lichen planus and immune balance of Th1/Th2 cells. Inflammation (2015) 38(5):1874–9. doi: 10.1007/s10753-015-0167-4 25825144

[55] Malekzadeh H Robati M Yousefimanesh H Ghafourian Boroujerdnia M Nadripour R. Salivary interferon gamma and interleukin-4 levels in patients suffering from oral lichen planus. Cell J (2015) 17(3):554–8. doi: 10.22074/cellj.2015.16 PMC4601877 26464828

[56] Pekiner FN Demirel GY Borahan MO Ozbayrak S. Cytokine profiles in serum of patients with oral lichen planus. Cytokine (2012) 60(3):701–6. doi: 10.1016/j.cyto.2012.08.007 22995209

[57] Karagouni EE Dotsika EN Sklavounou A. Alteration in peripheral blood mononuclear cell function and serum cytokines in oral lichen planus. J Oral Pathol Med (1994) 23(1):28–35. doi: 10.1111/j.1600-0714.1994.tb00250.x 8138978

[58] Simark-Mattsson C Bergenholtz G Jontell M Eklund C Seymour GJ Sugerman PB. Distribution of interleukin-2, -4, -10, tumour necrosis factor-alpha and transforming growth factor-beta mRNAs in oral lichen planus. Arch Oral Biol (1999) 44(6):499–507. doi: 10.1016/s0003-9969(99)00013-8 10401528

[59] Liu W Dan H Wang Z Jiang L Zhou Y Zhao M. IFN-gamma and IL-4 in saliva of patients with oral lichen planus: a study in an ethnic Chinese population. Inflammation (2009) 32(3):176–81. doi: 10.1007/s10753-009-9118-2 19370405

[60] Romagnani S Maggi E Liotta F Cosmi L Annunziato F. Properties and origin of human Th17 cells. Mol Immunol (2009) 47(1):3–7. doi: 10.1016/j.molimm.2008.12.019 19193443

[61] Xie S Ding L Xiong Z Zhu S. Implications of Th1 and Th17 cells in pathogenesis of oral lichen planus. J Huazhong Univ Sci Technol Med Sci (2012) 32(3):451–7. doi: 10.1007/s11596-012-0078-7 22684574

[62] Oppmann B Lesley R Blom B Timans JC Xu Y Hunte B. Novel p19 protein engages IL-12p40 to form a cytokine, IL-23, with biological activities similar as well as distinct from IL-12. Immunity (2000) 13(5):715–25. doi: 10.1016/s1074-7613(00)00070-4 11114383

[63] McGeachy MJ Cua DJ. Th17 cell differentiation: the long and winding road. Immunity (2008) 28(4):445–53. doi: 10.1016/j.immuni.2008.03.001 18400187

[64] Kreymborg K Etzensperger R Dumoutier L Haak S Rebollo A Buch T. IL-22 is expressed by Th17 cells in an IL-23-dependent fashion, but not required for the development of autoimmune encephalomyelitis. J Immunol (2007) 179(12):8098–104. doi: 10.4049/jimmunol.179.12.8098 18056351

[65] Piccinni MP Lombardelli L Logiodice F Tesi D Kullolli O Biagiotti R. Potential pathogenetic role of Th17, Th0, and Th2 cells in erosive and reticular oral lichen planus. Oral Dis (2014) 20(2):212–8. doi: 10.1111/odi.12094 23556506

[66] Lu R Zeng X Han Q Lin M Long L Dan H. Overexpression and selectively regulatory roles of IL-23/IL-17 axis in the lesions of oral lichen planus. Mediators Inflammation (2014) 2014:701094. doi: 10.1155/2014/701094 PMC4121042 25114378

[67] Josefowicz SZ Lu LF Rudensky AY. Regulatory T cells: mechanisms of differentiation and function. Annu Rev Immunol (2012) 30:531–64. doi: 10.1146/annurev.immunol.25.022106.141623 PMC6066374 22224781

[68] Tao XA Xia J Chen XB Wang H Dai YH Rhodus NL. FOXP3 T regulatory cells in lesions of oral lichen planus correlated with disease activity. Oral Dis (2010) 16(1):76–82. doi: 10.1111/j.1601-0825.2009.01608.x 19650850

[69] Mucida D Pino-Lagos K Kim G Nowak E Benson MJ Kronenberg M. Retinoic acid can directly promote TGF-beta-mediated Foxp3(+) treg cell conversion of naive T cells. Immunity (2009) 30(4):471–2. doi: 10.1016/j.immuni.2009.03.008 PMC2864308 19371709

[70] Ishigame H Zenewicz LA Sanjabi S Licona-Limón P Nakayama M Leonard WJ. Excessive Th1 responses due to the absence of TGF-β signaling cause autoimmune diabetes and dysregulated treg cell homeostasis. Proc Natl Acad Sci USA (2013) 110(17):6961–6. doi: 10.1073/pnas.1304498110 PMC3637710 23569233

[71] Collison LW Workman CJ Kuo TT Boyd K Wang Y Vignali KM. The inhibitory cytokine IL-35 contributes to regulatory T-cell function. Nature (2007) 450(7169):566–9. doi: 10.1038/nature06306 18033300

[72] Wolk K Haugen HS Xu W Witte E Waggie K Anderson M. IL-22 and IL-20 are key mediators of the epidermal alterations in psoriasis while IL-17 and IFN-gamma are not. J Mol Med (Berl) (2009) 87(5):523–36. doi: 10.1007/s00109-009-0457-0 19330474

[73] Boniface K Bernard FX Garcia M Gurney AL Lecron JC Morel F. IL-22 inhibits epidermal differentiation and induces proinflammatory gene expression and migration of human keratinocytes. J Immunol (2005) 174(6):3695–702. doi: 10.4049/jimmunol.174.6.3695 15749908

[74] Zotos D Coquet JM Zhang Y Light A D'Costa K Kallies A. IL-21 regulates germinal center b cell differentiation and proliferation through a b cell-intrinsic mechanism. J Exp Med (2010) 207(2):365–78. doi: 10.1084/jem.20091777 PMC2822601 20142430

[75] Tan YQ Li Q Zhang J Du GF Lu R Zhou G. Increased circulating CXCR5+ CD4+ T follicular helper-like cells in oral lichen planus. J Oral Pathol Med (2017) 46(9):803–9. doi: 10.1111/jop.12550 28122164

[76] Sanchez-Munoz F Dominguez-Lopez A Yamamoto-Furusho JK. Role of cytokines in inflammatory bowel disease. World J Gastroenterol (2008) 14(27):4280–8. doi: 10.3748/wjg.14.4280 PMC2731177 18666314

[77] Preshaw PM Taylor JJ. How has research into cytokine interactions and their role in driving immune responses impacted our understanding of periodontitis? J Clin Periodontol (2011) 38 Suppl 11:60–84. doi: 10.1111/j.1600-051X.2010.01671.x 21323705

[78] O'Shea JJ Murray PJ. Cytokine signaling modules in inflammatory responses. Immunity (2008) 28(4):477–87. doi: 10.1016/j.immuni.2008.03.002 PMC2782488 18400190

[79] Kumar DL Kumar PL James PF. Methylation-dependent and independent regulatory regions in the Na,K-ATPase alpha4 (Atp1a4) gene may impact its testis-specific expression. Gene (2016) 575(2 Pt 1):339–52. doi: 10.1016/j.gene.2015.09.003 PMC4662617 26343794

[80] Shan J Li S Wang C Liu L Wang X Zhu D. Expression and biological functions of the CCL5-CCR5 axis in oral lichen planus. Exp Dermatol (2019) 28(7):816–21. doi: 10.1111/exd.13946 31006151

[81] Ichimura M Hiratsuka K Ogura N Utsunomiya T Sakamaki H Kondoh T. Expression profile of chemokines and chemokine receptors in epithelial cell layers of oral lichen planus. J Oral Pathol Med (2006) 35(3):167–74. doi: 10.1111/j.1600-0714.2006.00402.x 16454813

[82] Lu R Zhang J Sun W Du G Zhou G. Inflammation-related cytokines in oral lichen planus: an overview. J Oral Pathol Med (2015) 44(1):1–14. doi: 10.1111/jop.12142 24329772

[83] Hasséus B Jontell M Brune M Johansson P Dahlgren UI. Langerhans cells and T cells in oral graft versus host disease and oral lichen planus. Scand J Immunol (2001) 54(5):516–24. doi: 10.1046/j.1365-3083.2001.00988.x 11696204

[84] Yamamoto T Osaki T. Characteristic cytokines generated by keratinocytes and mononuclear infiltrates in oral lichen planus. J Invest Dermatol (1995) 104(5):784–8. doi: 10.1111/1523-1747.ep12606990 7738356

[85] Du GH Qin XP Li Q Zhou YM Shen XM Tang GY. The high expression level of programmed death-1 ligand 2 in oral lichen planus and the possible costimulatory effect on human T cells. J Oral Pathol Med (2011) 40(7):525–32. doi: 10.1111/j.1600-0714.2011.01035.x 21457347

[86] Zhou G Zhang J Ren XW Hu JY Du GF Xu XY. Increased B7-H1 expression on peripheral blood T cells in oral lichen planus correlated with disease severity. J Clin Immunol (2012) 32(4):794–801. doi: 10.1007/s10875-012-9683-2 22430646

[87] Tao XA Li CY Rhodus NL Xia J Yang XP Cheng B. Simultaneous detection of IFN-gamma and IL-4 in lesional tissues and whole unstimulated saliva from patients with oral lichen planus. J Oral Pathol Med (2008) 37(2):83–7. doi: 10.1111/j.1600-0714.2007.00593.x 18197852

[88] Gu GM Martin MD Darveau RP Truelove E Epstein J. Oral and serum IL-6 levels in oral lichen planus patients. Oral Surg Oral Med Oral Pathol Oral Radiol Endod (2004) 98(6):673–8. doi: 10.1016/j.tripleo.2004.05.006 15583539

[89] Rhodus NL Cheng B Bowles W Myers S Miller L Ondrey F. Proinflammatory cytokine levels in saliva before and after treatment of (erosive) oral lichen planus with dexamethasone. Oral Dis (2006) 12(2):112–6. doi: 10.1111/j.1601-0825.2005.01165.x 16476030

[90] Kho HS Chang JY Kim YY Kim Y. MUC1 and toll-like receptor-2 expression in burning mouth syndrome and oral lichen planus. Arch Oral Biol (2013) 58(7):837–42. doi: 10.1016/j.archoralbio.2013.01.008 23411403

[91] Younes F Quartey EL Kiguwa S Partridge M. Expression of TNF and the 55-kDa TNF receptor in epidermis, oral mucosa, lichen planus and squamous cell carcinoma. Oral Dis (1996) 2(1):25–31. doi: 10.1111/j.1601-0825.1996.tb00199.x 8957934

[92] Karatsaidis A Hayashi K Schreurs O Helgeland K Schenck K. Survival signalling in keratinocytes of erythematous oral lichen planus. J Oral Pathol Med (2007) 36(4):215–22. doi: 10.1111/j.1600-0714.2007.00519.x 17391299

[93] Thongprasom K Dhanuthai K Sarideechaigul W Chaiyarit P Chaimusig M. Expression of TNF-alpha in oral lichen planus treated with fluocinolone acetonide 0.1%. J Oral Pathol Med (2006) 35(3):161–6. doi: 10.1111/j.1600-0714.2006.00392.x 16454812

[94] Alrashdan MS Cirillo N McCullough M. Oral lichen planus: a literature review and update. Arch Dermatol Res (2016) 308(8):539–51. doi: 10.1007/s00403-016-1667-2 27349424

[95] Khan A Farah CS Savage NW Walsh LJ Harbrow DJ Sugerman PB. Th1 cytokines in oral lichen planus. J Oral Pathol Med (2003) 32(2):77–83. doi: 10.1034/j.1600-0714.2003.00077.x 12542829

[96] Zhou XJ Sugerman PB Savage NW Walsh LJ. Matrix metalloproteinases and their inhibitors in oral lichen planus. J Cutan Pathol (2001) 28(2):72–82. doi: 10.1034/j.1600-0560.2001.280203.x 11168755

[97] Juneja M Mahajan S Rao NN George T Boaz K. Histochemical analysis of pathological alterations in oral lichen planus and oral lichenoid lesions. J Oral Sci (2006) 48(4):185–93. doi: 10.2334/josnusd.48.185 17220615

[98] Peng Q Yang JY Zhou G. Emerging functions and clinical applications of exosomes in human oral diseases. Cell Biosci (2020) 10:68. doi: 10.1186/s13578-020-00424-0 32489584 PMC7245751

[99] Xu AT Lu JT Ran ZH Zheng Q. Exosome in intestinal mucosal immunity. J Gastroenterol Hepatol (2016) 31(10):1694–9. doi: 10.1111/jgh.13413 27061439

[100] Greening DW Gopal SK Xu R Simpson RJ Chen W. Exosomes and their roles in immune regulation and cancer. Semin Cell Dev Biol (2015) 40:72–81. doi: 10.1016/j.semcdb.2015.02.009 25724562

[101] Huang A Dong J Li S Wang C Ding H Li H. Exosomal transfer of vasorin expressed in hepatocellular carcinoma cells promotes migration of human umbilical vein endothelial cells. Int J Biol Sci (2015) 11(8):961–9. doi: 10.7150/ijbs.11943 PMC4495413 26157350

[102] Peng Q Zhang J Zhou G. Differentially circulating exosomal microRNAs expression profiling in oral lichen planus. Am J Transl Res (2018) 10(9):2848–58. doi: 10.1111/jgh.13413 PMC6176222 30323871

[103] Peng Q Zhang J Zhou G. Circulating exosomes regulate T-cell-mediated inflammatory response in oral lichen planus. J Oral Pathol Med (2019) 48(2):143–50. doi: 10.1111/jop.12804 30447107

[104] Byun JS Hong SH Choi JK Jung JK Lee HJ. Diagnostic profiling of salivary exosomal microRNAs in oral lichen planus patients. Oral Dis (2015) 21(8):987–93. doi: 10.1111/odi.12374 26389700

[105] Jablonska E Garley M Surazynski A Grubczak K Iwaniuk A Borys J. Neutrophil extracellular traps (NETs) formation induced by TGF-β in oral lichen planus - possible implications for the development of oral cancer. Immunobiology (2020) 225(2):151901. doi: 10.1016/j.imbio.2019.151901 31882256

[106] Kirchner T Hermann E Möller S Klinger M Solbach W Laskay T. Flavonoids and 5-aminosalicylic acid inhibit the formation of neutrophil extracellular traps. Mediators Inflammation (2013) 2013:710239. doi: 10.1155/2013/710239 PMC3871909 24381411

[107] Carrozzo M Uboldi de Capei M Dametto E Fasano ME Arduino P Broccoletti R. Tumor necrosis factor-alpha and interferon-gamma polymorphisms contribute to susceptibility to oral lichen planus. J Invest Dermatol (2004) 122(1):87–94. doi: 10.1046/j.0022-202X.2003.22108.x 14962095

[108] Siponen M Huuskonen L Kallio-Pulkkinen S Nieminen P Salo T. Topical tacrolimus, triamcinolone acetonide, and placebo in oral lichen planus: a pilot randomized controlled trial. Oral Dis (2017) 23(5):660–8. doi: 10.1111/odi.12653 28168769

[109] Singh AR Rai A Aftab M Jain S Singh M. Efficacy of steroidal vs non-steroidal agents in oral lichen planus: a randomised, open-label study. J Laryngol Otol (2017) 131(1):69–76. doi: 10.1017/S0022215116009658 27917729

[110] Sivaraman S Santham K Nelson A Laliytha B Azhalvel P Deepak JH. A randomized triple-blind clinical trial to compare the effectiveness of topical triamcinolone acetonate (0.1%), clobetasol propionate (0.05%), and tacrolimus orabase (0.03%) in the management of oral lichen planus. J Pharm Bioallied Sci (2016) 8(Suppl 1):S86–9. doi: 10.4103/0975-7406.191976 PMC5074049 27829754

[111] Vohra S Singal A Sharma SB. Clinical and serological efficacy of topical calcineurin inhibitors in oral lichen planus: a prospective randomized controlled trial. Int J Dermatol (2016) 55(1):101–5. doi: 10.1111/ijd.12887 26147635

[112] Hettiarachchi PVKS Hettiarachchi RM Jayasinghe RD Sitheeque M. Comparison of topical tacrolimus and clobetasol in the management of symptomatic oral lichen planus: A double-blinded, randomized clinical trial in Sri Lanka. J Investig Clin Dent (2017) 8(4):1166–1176. doi: 10.1111/jicd.12237 27633647

[113] Samimi M Le Gouge A Boralevi F Passeron T Pascal F Bernard P. Topical rapamycin versus betamethasone dipropionate ointment for treating oral erosive lichen planus: a randomized, double-blind, controlled study. J Eur Acad Dermatol Venereol (2020) 34(10):2384–91. doi: 10.1111/jdv.16324 32128907

[114] Ezzatt OM Helmy IM. Topical pimecrolimus versus betamethasone for oral lichen planus: a randomized clinical trial. Clin Oral Investig (2019) 23(2):947–56. doi: 10.1007/s00784-018-2519-6 29909565

[115] Carbone M Conrotto D Carrozzo M Broccoletti R Gandolfo S Scully C. Topical corticosteroids in association with miconazole and chlorhexidine in the long-term management of atrophic-erosive oral lichen planus: a placebo-controlled and comparative study between clobetasol and fluocinonide. Oral Dis (1999) 5(1):44–9. doi: 10.1111/j.1601-0825.1999.tb00063.x 10218041

[116] Al-Hashimi I Schifter M Lockhart PB Wray D Brennan M Migliorati CA. Oral lichen planus and oral lichenoid lesions: diagnostic and therapeutic considerations. Oral Surg Oral Med Oral Pathol Oral Radiol Endod (2007) 103 Suppl:S25.e1–12. doi: 10.1016/j.tripleo.2006.11.001 17261375

[117] Riaz H Shakeel A Shaheen MA Jaa K. Efficacy of pimecrolimus cream and triamcinolone acetonide paste in the treatment of symptomatic oral lichen planus. Med Forum Monthly (2017) 28(12):76–80. doi: 10.1111/j.1600-0714.2009.00805.x

[118] Thongprasom K Chaimusig M Korkij W Sererat T Luangjarmekorn L Rojwattanasirivej S. A randomized-controlled trial to compare topical cyclosporin with triamcinolone acetonide for the treatment of oral lichen planus. J Oral Pathol Med (2007) 36(3):142–6. doi: 10.1111/j.1600-0714.2007.00510.x 17305635

[119] Yoke PC Tin GB Kim MJ Rajaseharan A Ahmed S Thongprasom K. A randomized controlled trial to compare steroid with cyclosporine for the topical treatment of oral lichen planus. Oral Surg Oral Med Oral Pathol Oral Radiol Endodontoll (2006) 102(1):47–55. doi: 10.1016/j.tripleo.2005.09.006 16831672

[120] Conrotto D Carbone M Carrozzo M Arduino P Broccoletti R Pentenero M. Ciclosporin vs. clobetasol in the topical management of atrophic and erosive oral lichen planus: a double-blind, randomized controlled trial. Br J Dermatol (2006) 154(1):139–45. doi: 10.1111/j.1365-2133.2005.06920.x 16403107

[121] Heffernan MP Smith DI Bentley D Tabacchi M Graves JE. A single-center, open-label, prospective pilot study of subcutaneous efalizumab for oral erosive lichen planus. J Drugs Dermatol (2007) 6(3):310–4. doi: 10.1111/jicd.12237 17373193

[122] Wu Y Zhou G Zeng H Xiong CR Lin M Zhou HM. A randomized double-blind, positive-control trial of topical thalidomide in erosive oral lichen planus. Oral Surg Oral Med Oral Pathol Oral Radiol Endodontoll (2010) 110(2):188–95. doi: 10.1016/j.tripleo.2010.03.034 20659697

[123] Samiee N Zenuz AT Shokri J Mehdipour M. Treatment of oral lichen planus with mucoadhesive mycophenolate mofetil patch; a randomized clinical trial. Clin Exp Dental Res (2020) 6(5):506–11. doi: 10.1002/cre2.302 PMC7545225 32592335

[124] Nasr MM Ebrahim HM Khattab FM Marei AM. Bacillus calmette-guerin, polysaccharide nucleic acid in the treatment of cutaneous and oral lichen planus. Dermatol Ther (2018) 31(3):e12591. doi: 10.1111/dth.12591 29405515

[125] Xiong C Li Q Lin M Li X Meng W Wu Y. The efficacy of topical intralesional BCG-PSN injection in the treatment of erosive oral lichen planus: a randomized controlled trial. J Oral Pathol Med (2009) 38(7):551–8. doi: 10.1111/j.1600-0714.2009.00796.x 19486267

[126] Yuan P Qiu X Ye L Hou F Liang Y Jiang H. Efficacy of topical administration for oral lichen planus: A network meta-analysis. Oral Dis (2022) 28(3):670–81. doi: 10.1111/odi.13790 33529456

[127] Sun SL Liu JJ Zhong B Wang JK Jin X Xu H. Topical calcineurin inhibitors in the treatment of oral lichen planus: a systematic review and meta-analysis. Br J Dermatol (2019) 181(6):1166–76. doi: 10.1111/bjd.17898 30903622

[128] Youngnak-Piboonratanakit P Dhanuthai K Thongprasom K Luckprom P Sarideechaigul W Luangjarmekorn L. Expression of IFN-gamma before and after treatment of oral lichen planus with 0.1% fluocinolone acetonide in orabase. J Oral Pathol Med (2009) 38(9):689–94. doi: 10.1111/j.1600-0714.2009.00805.x 19614863

[129] Crabtree GR Schreiber SL. SnapShot: Ca2+-calcineurin-NFAT signaling. Cell (2009) 138(1):210, 210.e1. doi: 10.1016/j.cell.2009.06.026 19596245 PMC2958059

[130] Azzi JR Sayegh MH Mallat SG. Calcineurin inhibitors: 40 years later, can't live without. . J Immunol (2013) 191(12):5785–91. doi: 10.4049/jimmunol.1390055 24319282

[131] Zhang Y Lin M Zhang S Wang Z Jiang L Shen J. NF-kappaB-dependent cytokines in saliva and serum from patients with oral lichen planus: a study in an ethnic Chinese population. Cytokine (2008) 41(2):144–9. doi: 10.1016/j.cyto.2007.11.004 18222093

[132] Gonzalez-Moles MA Ruiz-Avila I Rodriguez-Archilla A Morales-Garcia P Mesa-Aguado F Bascones-Martinez A. Treatment of severe erosive gingival lesions by topical application of clobetasol propionate in custom trays. Oral Surg Oral Med Oral Pathol Oral Radiol Endod (2003) 95(6):688–92. doi: 10.1067/moe.2003.139 12789149

[133] Eisen D Carrozzo M Bagan Sebastian JV Thongprasom K. Number V oral lichen planus: clinical features and management. Oral Dis (2005) 11(6):338–49. doi: 10.1111/j.1601-0825.2005.01142.x 16269024

[134] Ibrahim SS Hazzaa HH. Topical pimecrolimus effect on fas inducing apoptosis in oral lichen planus: a clinical immunohistochemical study. J Oral Pathol Med (2012) 41(4):315–21. doi: 10.1111/j.1600-0714.2011.01099.x 22085391

[135] Miroux Céline Morales O Ghazal K Othman SB De Launoit Y Pancré Véronique. *In vitro* effects of cyclosporine a and tacrolimus on regulatory t-cell proliferation and function. Transplantation (2013) 94(2):123–31. doi: 10.1016/j.tripleo.2010.03.034 22743548

[136] Ge Y Xu Y Sun W Man Z Zhu L Xia X. The molecular mechanisms of the effect of dexamethasone and cyclosporin a on TLR4 /NF-κB signaling pathway activation in oral lichen planus. Gene (2012) 508(2):157–64. doi: 10.1016/j.gene.2012.07.045 22903029

[137] Franks ME Macpherson GR Figg WD. Thalidomide. Lancet (2004) 363(9423):1802–11. doi: 10.1016/S0140-6736(04)16308-3 15172781

[138] Frieling U Bonsmann G Schwarz T Luger TA Beissert S. Treatment of severe lichen planus with mycophenolate mofetil. J Am Acad Dermatol (2003) 49(6):1063–6. doi: 10.1016/s0190-9622(03)02111-x 14639385

[139] Olejarz W Bryk D Zapolska-Downar D. Mycophenolate mofetil–a new atheropreventive drug? Acta Pol Pharm (2014) 71(3):353–61. doi: 10.1517/14728214.2011.528390 25265813

[140] Elad S Epstein JB von Bültzingslöwen I Drucker S Tzach R Yarom N. Topical immunomodulators for management of oral mucosal conditions, a systematic review; part II: miscellaneous agents. Expert Opin Emerg Drugs (2011) 16(1):183–202. doi: 10.1517/14728214.2011.528390 21244328

[141] Guenther LC Kunynetz R Lynde CW Sibbald RG Toole J Vender R. Acitretin use in dermatology. J Cutan Med Surg (2017) 21(3_suppl):2S–12S. doi: 10.1177/1203475417733414 28952335

[142] Leonardi CL. Efalizumab: an overview. J Am Acad Dermatol (2003) 49(2 Suppl):S98–104. doi: 10.1016/s0190-9622(03)01141-1 12894132

[143] Gisondi P Girolomoni G. Biologic therapies in psoriasis: a new therapeutic approach. Autoimmun Rev (2007) 6(8):515–9. doi: 10.1016/j.autrev.2006.12.002 17854741

[144] Deng Y Chen Y Chen X Li Y Zhou L. A comparative study on the effect of BCG-PSN and thymopeptides on T-lymphocyte subsets of normal and immunosuppressed mice. J Huazhong Univ Sci Technol Med Sci (2003) 23(4):339–43. doi: 10.1007/BF02829412 15015630

[145] Giri PK Schorey JS. Exosomes derived from m. bovis BCG infected macrophages activate antigen-specific CD4+ and CD8+ T cells *in vitro* and *in vivo* . PloS One (2008) 3(6):e2461. doi: 10.1371/journal.pone.0002461 18560543 PMC2413420

[146] Zhou G Fan MW Liu JY. Regulation of BCG-polysaccharide nucleic acid on Thl/Th2 cytokines from peripheral blood mononuclear cells in oral lichen planus. Chin J Dent Res (2004) 7:5–10. doi: 10.1007/BF02829412

[147] Si L. Observation of the treatment of oral lichen planus in 40 cases with integrated Chinese and Western medicine. Guo Yi Lun Tan (2017) 32(04):47–8. doi: 10.13913/j.cnki.41-1110/r.2017.04.024

[148] Hao X Guan X. Study on the medication regularity of oral lichen planus treated with TCM. Zhongguo Min Jian Liao Fa (2020) 28(22):55–7. doi: 10.19621/j.cnki.11-3555/r.2020.2224

[149] Boisnic S Frances C Branchet MC Szpirglas H Le Charpentier Y. Immunohistochemical study of oral lesions of lichen planus: diagnostic and pathophysiologic aspects. Oral Surg Oral Med Oral Pathol (1990) 70(4):462–5. doi: 10.1016/0030-4220(90)90211-a 1977114

[150] Pang J Bu J. Immunoregulation effect of traditional Chinese medicine treatment on patients with oral lichen planus. Zhonghua Kou Qiang Yi Xue Za Zhi (1998) 33(1):48–9. 11774680

